# Multiple brown tumors as a result of hyperparathyroidism in chronic renal disease: a case report

**DOI:** 10.11604/pamj.2017.26.144.12026

**Published:** 2017-03-14

**Authors:** Moncef Sellami, Yosr Chaabouni

**Affiliations:** 1 Department of Otorhinolaryngology-Head and Neck Surgery Habib Bourguiba University Hospital, Sfax, Tunisia; 2 Sfax Medical School,University of Sfax, Sfax, Tunisia; 3 Department of Nephrology, Research Unit UR12ES14. Hedi Chaker University Hospital, Sfax, Tunis

**Keywords:** Brown tumor, chronic renal disease, hyperparathyroidism

## Image in medicine

The brown tumor or osteitis fibrosacystica is a benign bone lesion that caused by hyperparathyroidism. This complication has been decreased by diagnosis and successful treatment of hyperparathyroidism. The brown tumor of hyperparathyroidism results from a metabolic disorder that affects long bones, ribs, and pelvis. The maxillary involvement is rare. Parathyroidectomyis a good choice for patients with high parathyroid hormone (PTH) levels and diffuse brown tumor leading to a gradual decrease in the maxillary tumors. We report the case of a 50-year-old woman with chronicrenal failure presented with one-year history of slowly enlarging mass of the left cheek after 17 years of maintenance hemodialysis. The physical examination revealed a mass of the left jaw and the infraorbital region associated with a left exophtalmia. The mass was soft in consistency and tender on palpation. Her serum PTH concentration was 4830 pg/ml and her calcemia was normal. The computed tomography (CT) of the facial skeleton showed multiple osteolytic lesions of the maxillary sinuses and mandibular bone suggestive of brown tumors. The expansive lesion of the left maxillary bone found an extensive involvement of the left maxilla and the maxillary sinus. The patient underwent a subtotal parathyroidectomy. The PTH concentration taken 1 hour after the surgery confirmed a level within the normal range. At 12-month follow-up, there was an improvement of the exophtalmia and partial regression of the tumors.

**Figure 1:**
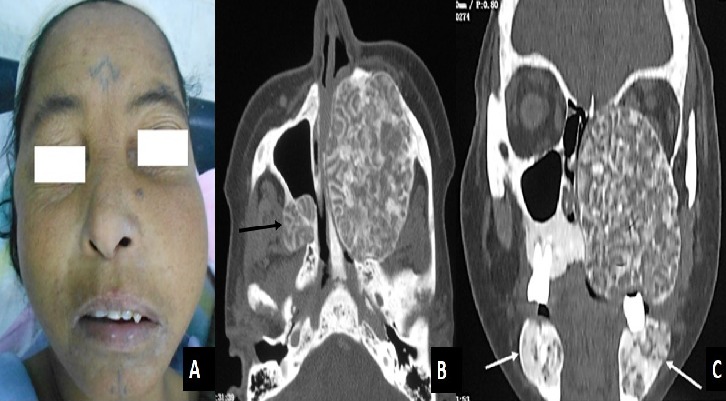
(A) swelling in the left cheek and the infraorbital region; (B ) axial CT scan image shows a destructive lesion of the left maxillary sinus with involvement associated to a lesion of the posterior wall of the right maxillary sinus (arrow); (C) coronal CT scan image shows an extensive involvement of the left maxilla and maxillary sinus associated to mandibular tumors (arrow)

